# Supplementary material for: Identification and functional analysis of non-coding regulatory small RNA FenSr3 in Bacillus amyloliquefaciens LPB-18
Source: PeerJ. 2023 May 15;11:e15236. doi: 10.7717/peerj.15236 (PMC10194069; doi:10.7717/peerj.15236)
Supplement: Supplemental Information 4 [file peerj-11-15236-s004.zip › KO/CK-vs-T1_map/map05100.html]

KEGG PATHWAY: Bacterial invasion of epithelial cells - Reference pathway


|  |  |
| --- | --- |
| **Bacterial invasion of epithelial cells - Reference pathway** |  |

[
Pathway menu
| Organism menu
| Pathway entry
| Show description
| User data mapping
]

Many pathogenic bacteria can invade phagocytic and non-phagocytic cells and colonize them intracellularly, then become disseminated to other cells. Invasive bacteria induce their own uptake by non-phagocytic host cells (e.g. epithelial cells) using two mechanisms referred to as zipper model and trigger model. Listeria, Staphylococcus, Streptococcus, and Yersinia are examples of bacteria that enter using the zipper model. These bacteria express proteins on their surfaces that interact with cellular receptors, initiating signalling cascades that result in close apposition of the cellular membrane around the entering bacteria. Shigella and Salmonella are the examples of bacteria entering cells using the trigger model. These bacteria use type III secretion systems to inject protein effectors that interact with the actin cytoskeleton.

|  |  |  |
| --- | --- | --- |
| Reference pathway | 184% 150% 122% 100% 82% 67% 55% | 图片下载 |
